# Validation of Azure Kinect for upper limb kinematic assessment after arthroscopic rotator cuff repair: task-dependent accuracy and postoperative overestimation

**DOI:** 10.3389/fbioe.2026.1927386

**Published:** 2026-09-22

**Authors:** Yu-fei Chen, Chuang-yu Zhan, Ling-jie Zhang, Yue-xin Zhang, Yun-ru Ma

**Affiliations:** 1 Department of the First School of Clinical Medicine, The First Affiliated Hospital of Fujian Medical University, Fuzhou, China; 2 Department of Rehabilitation Medicine, School of Health, Fujian Medical University, Fuzhou, China; 3 College of Computer and Cyber Security, Fujian Normal University, Fuzhou, China; 4 Fujian Provincial Key Laboratory of Network Security and Cryptology, Fujian Normal University, Fuzhou, China

**Keywords:** arthroscopic repair, Azure Kinect, depth sensor, inertial measurement unit, markerless motion analysis, rotator cuff tear, upper limb movement analysis

## Abstract

**Introduction:**

Arthroscopic rotator cuff repair is the standard treatment for rotator cuff tears (RCTs), yet postoperative functional evaluation continues to rely heavily on subjective clinical scales, with limited objective kinematic assessment. This study evaluated the concurrent validity of an Azure Kinect-based markerless system against a reference inertial measurement unit (IMU) system for upper limb kinematic assessment in patients undergoing RCT repair, and explored its postoperative clinical utility.

**Methods:**

Twelve patients with unilateral full-thickness RCTs were assessed preoperatively and at four months postoperatively. Participants performed six functional activities of daily living tasks and 3D shoulder range-of-motion (ROM) tests, with kinematic data synchronously collected by both systems. Inter-system agreement was evaluated using intraclass correlation coefficients (ICC2,5), Pearson’s r, Lin’s concordance correlation coefficient (CCC), Bland-Altman analysis, waveform similarity metrics (coefficient of multiple correlation - CMC), and error metrics (root mean square error-RMSE, mean absolute error-MAE, standard error of measurement-SEM).

**Results:**

During functional tasks, Azure Kinect demonstrated good agreement with IMUs for sagittal-plane movements (ICC2,5 = 0.708 − 0.907 for shoulder flexion; 0.804 − 0.846 for elbow flexion) with good to excellent waveform consistency (CMC > 0.75; CCC > 0.70), but substantially lower agreement and waveform similarity in coronal and horizontal planes. Bland–Altman analysis showed relatively small sagittal-plane errors (SEM < 15°) but larger biases in non-sagittal planes, with proportional bias detected for most joint planes and tasks (p < 0.05). For 3D shoulder ROM assessment, good agreement was observed for flexion (ICC2,5 = 0.837), abduction (ICC2,5 = 0.729), and rotation (ICC2,5 = 0.735). No significant preoperative inter-system differences were found. Postoperatively, Azure Kinect significantly overestimated flexion (137.9 ± 13.5° vs. 97.1 ± 25.1°, p = 0.002) and external rotation (48.6 ± 14.6° vs. 31.0 ± 14.4°, p = 0.038). Both systems detected significant flexion changes pre-to-post, but neither detected changes in abduction or rotation.

**Discussion:**

These preliminary findings indicate task- and plane-dependent validity for the Azure Kinect, with good sagittal performance but substantial errors in multi-planar motions and postoperative overestimation. The system may serve as a screening tool for tracking postoperative functional recovery, but its current accuracy is insufficient for standalone surgical outcome assessment. Results should be interpreted with caution given the small sample size and confirmed in larger cohorts.

## 1 Introduction

Rotator cuff tear (RCT) is among the most prevalent musculoskeletal conditions, with a reported prevalence of 22.1% in the general population and a significant age-related increase in incidence (Jeong et al., 2017; Minagawa et al., 2013). Given that RCT restricts shoulder mobility and induces muscle weakness, patients’ activities of daily living (ADL) and quality of life are profoundly affected (Cho et al., 2015). Arthroscopic rotator cuff repair has become a reliable surgical treatment over the past 30 years (Mancini et al., 2023), yet postoperative functional movement assessment remains heavily reliant on subjective rating scales (Ashton et al., 2020) and surgeon assigned visual assessment (Burkhart et al., 2020). While these tools offer clinical convenience, their drawbacks, including assessment bias, insufficient sensitivity to subtle functional changes, and cross-cultural validity concerns, have been well documented. These limitations have motivated a growing interest in objective, quantitative movement analysis as a complementary approach to traditional outcome measures.

In this context, optoelectronic marker-based motion capture systems are widely considered as the gold standard for quantifying upper limb kinematics. These systems provide high-precision, reliable kinematic data and have been successfully applied to analyze movement deficits in patients with RCTs (Fritz et al., 2017; Nyőgér et al., 2025; Vidt et al., 2016). However, their clinical application is constrained by high equipment costs, extensive preparation time, reliance on specialized laboratory settings, and the need for trained operators. Additionally, skin-attached reflective markers may cause self-consciousness in patients, potentially altering natural movement patterns and limiting ecological validity (Adlou et al., 2025).

Wearable inertial measurement units (IMUs) have emerged as an alternative that expands the scope of clinical application by enabling motion capture unconstrained by laboratory environments. By measuring shoulder kinematics during actual ADLs, IMUs can reveal whether clinic-measured improvements translate into meaningful functional gains in real-world settings (Chapman et al., 2023; Kwak et al., 2020; Thakkar et al., 2026). Furthermore, their wearable, unsupervised nature supports remote rehabilitation monitoring and pathological condition recognition, enabling clinicians to track treatment outcome without frequent in-person visits (Chen et al., 2026). Nevertheless, IMU systems require multiple body-mounted sensors, complex calibration procedures (Fang et al., 2023), and may cause discomfort during testing, all of which reduce their feasibility in routine clinical scenarios.

The shared limitations of optoelectronic and IMU motion capture systems motivate the development of markerless movement tracking that can quantify 3D kinematics without any body-worn devices, thereby preserving undisturbed movement patterns while enabling practical clinical use. Depth-camera–based markerless motion capture systems have emerged as a potential alternative for clinical movement analysis. The Microsoft Azure Kinect sensor uses RGB-D imaging to estimate human skeletal landmarks without reflective markers or wearable devices. Because of its portability, low cost, and ease of operation, Azure Kinect has attracted attention as a potential tool for clinical motion assessment. Previous studies have evaluated the reliability and validity of Azure Kinect in gait analysis (Cui et al., 2026; Lin et al., 2025), balance assessment (Tapanya et al., 2023), rehabilitation training (Dajime and Zhang, 2023), as well as upper limb movement assessment (Lauman et al., 2025; Özsoy et al., 2022). However, most studies were conducted in healthy populations or focused primarily on simple shoulder range-of-motion (ROM) measurements. Evidence regarding its validity for evaluating complex upper limb functional movements in clinical populations after rotator cuff repair remains limited. To the best of the authors’ knowledge, no study has examined whether the Azure Kinect-based motion capture system can detect arthroscopic surgery-related kinematic changes.

Therefore, the primary aim of this exploratory study was to evaluate the concurrent validity of the Azure Kinect system for measuring upper limb kinematics during functional tasks in patients with RCTs by comparing it with a reference IMU-based motion capture system. The secondary aim was to investigate whether Azure Kinect could serve as a feasible tool for postoperative shoulder function assessment after arthroscopic rotator cuff repair. We hypothesized that Azure Kinect would demonstrate acceptable agreement with the IMU system for measuring sagittal-plane movements and shoulder range of motion, but lower agreement for multi-planar movements.

## 2 Methodology

### 2.1 Study design and participants

This prospective observational study was approved by the Institutional Review Board of The First Affiliated Hospital of Fujian Medical University (approval number: 2023455). Written informed consent was obtained from all participants prior to enrollment. The study was prospectively registered in the Clinical Trial Registry (registration number: ChiCTR2300076496). Patients diagnosed with unilateral RCTs who underwent arthroscopic rotator cuff repair between October 2023 and March 2025 were recruited from the Department of Orthopedics at The First Affiliated Hospital of Fujian Medical University.

A total of 12 patients (four males and eight females) participated. The mean (±standard deviation) characteristics of the participants were: age 54.1 ± 7.7 years, height 163.6 ± 8.0 cm, mass 62.5 ± 10.4 kg, BMI 23.2 ± 2.7 kg/m^2^. All patients had unilateral full-thickness RCTs, classified as small-to-medium tears (<3 cm) according to the DeOrio and Cofield classification (DeOrio and Cofield, 1984).

Patients were included if they were adults with unilateral full-thickness RCTs undergoing arthroscopic repair and able to perform the required functional tasks. Patients were excluded if they had previous surgery on the affected shoulder, severe shoulder stiffness, neurological or musculoskeletal disorders affecting upper limb movement, or incomplete follow-up data. Assessments were performed preoperatively and at a 4-month postoperative follow-up. This time point was chosen because most patients have completed early rehabilitation and regained sufficient functional mobility for movement assessment.

### 2.2 Experimental protocol

Upper limb kinematics were recorded simultaneously using an IMU-based motion capture system (Portable Lab, Noraxon Inc., Scottsdale, AZ, United States) and a Microsoft Azure Kinect depth camera (Microsoft Corp., Redmond, WA, United States). Both systems were started manually by a single operator as closely as possible. The IMU system was selected as the reference comparator for this study based on clinical practicality and established validity. The IMU system applied in this study has been validated against optoelectronic systems for upper limb kinematic assessment, although its measurement accuracy varies by movement plane. For shoulder flexion/extension, the system shows high correlation with reference systems (r = 0.98–0.99) with standard errors of estimate (SEE) of 8.2°–9.4°. However, larger errors have been reported for shoulder abduction (SEE = 31.7°–32.8°, r = 0.57–0.70) and elbow flexion/extension (SEE = 23.7°–23.8°, r = 0.67–0.93) (Bartoszek et al., 2022). Comparison with goniometry has also revealed mean biases ranging from −28.3° to 5.3° for shoulder abduction and −22.5° to 12.2° for shoulder rotation (Yoon, 2017). These known error margins should be considered when interpreting the IMU-derived reference values in this study.

For the IMU-based system, ten wearable sensors were attached to the participant’s head, hands, forearms, upper arms, sacrum, and the C7 and L1/T12 spinal levels by using elastic straps. Each sensor contained a triaxial accelerometer (±16 g), gyroscope (±2000°/s), and magnetometer (±4,900 µT), enabling measurement of linear acceleration, angular velocity, and magnetic field data along the x, y, and z axes. Motion signals were recorded at a sampling frequency of 100 Hz. Prior to each testing session, the IMU system underwent a zero-calibration procedure to reset gyros of all applied sensors. Then, a static posture calibration was conducted to create each participant’s upper limb segmental coordinate systems. Sensors were placed by a trained investigator to ensure consistent placement across participants.

The Azure Kinect sensor was positioned approximately 2 m in front of the participant and 0.8 m above the floor, with the height fixed for all participants. The camera placement was determined based on preliminary testing to minimize self-occlusion and ensure optimal skeletal tracking during upper limb movements. All testing sessions were conducted in the same consulting room under consistent indoor lighting, with a plain, non-reflective wall background behind the participant. Participants were instructed to wear close-fitting clothing and stand face to the camera for all tasks. The sensor’s built-in automatic calibration was used. No additional manual distance calibration was performed, consistent with the device’s intended plug-and-use design for clinical applications.

A C# program based on the Microsoft Azure Kinect Software Development Kit (SDK) was applied to track and record the three-dimensional coordinates of 32 body landmarks in the camera’s global coordinate system (in millimeters) with a sampling frequency of approximately 30 Hz, as shown in Figure 1. In this coordinate system, the x-axis represents the mediolateral direction, the y-axis represents the vertical direction, and the z-axis represents the anteroposterior direction. All landmark trajectories were exported as CSV files for further joint angle computation. The data acquisition process is depicted in Figure 2A.

**FIGURE 1:**
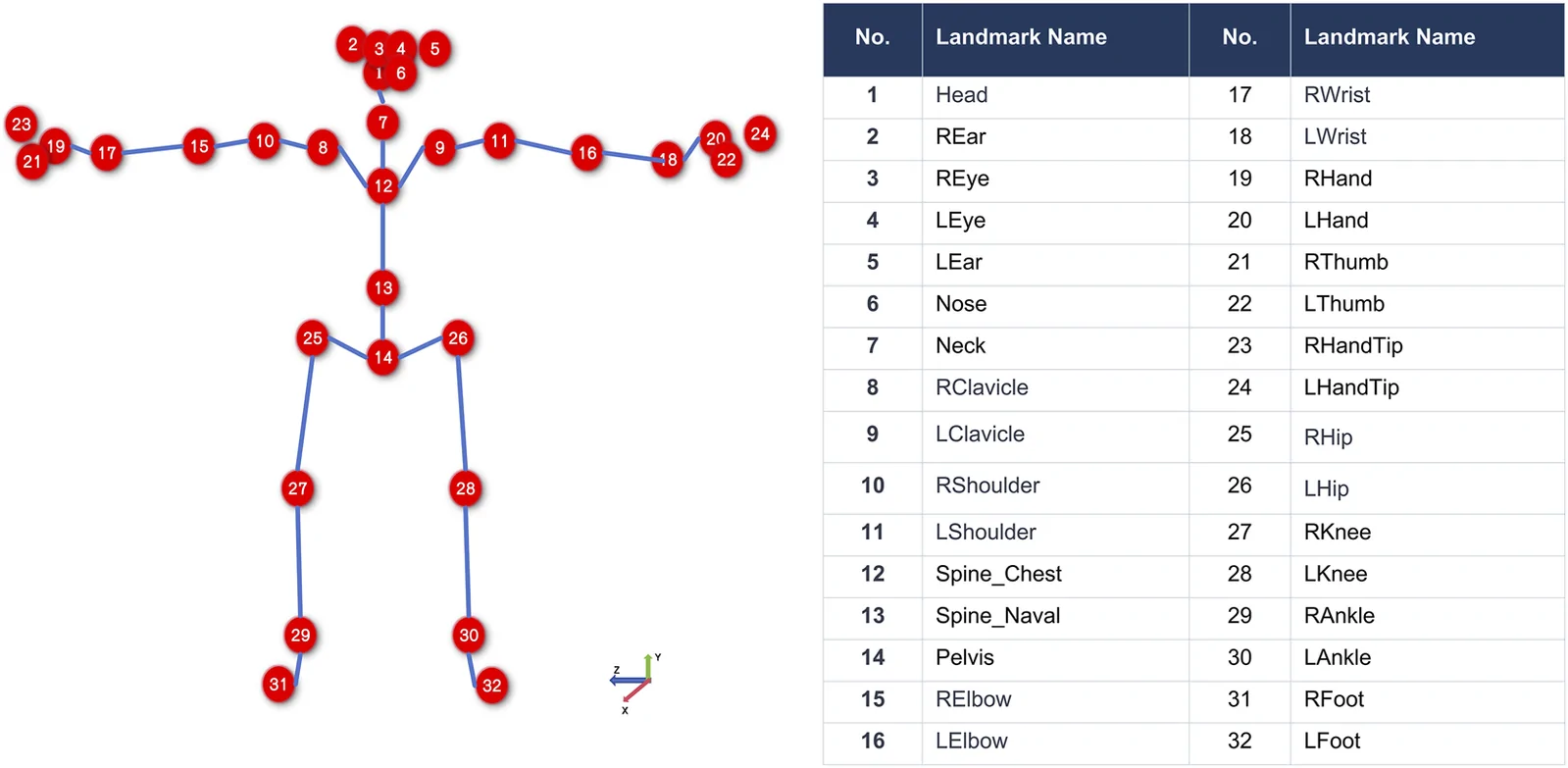
The 32 body landmarks tracked by the Azure Kinect Body Tracking SDK.

**FIGURE 2:**
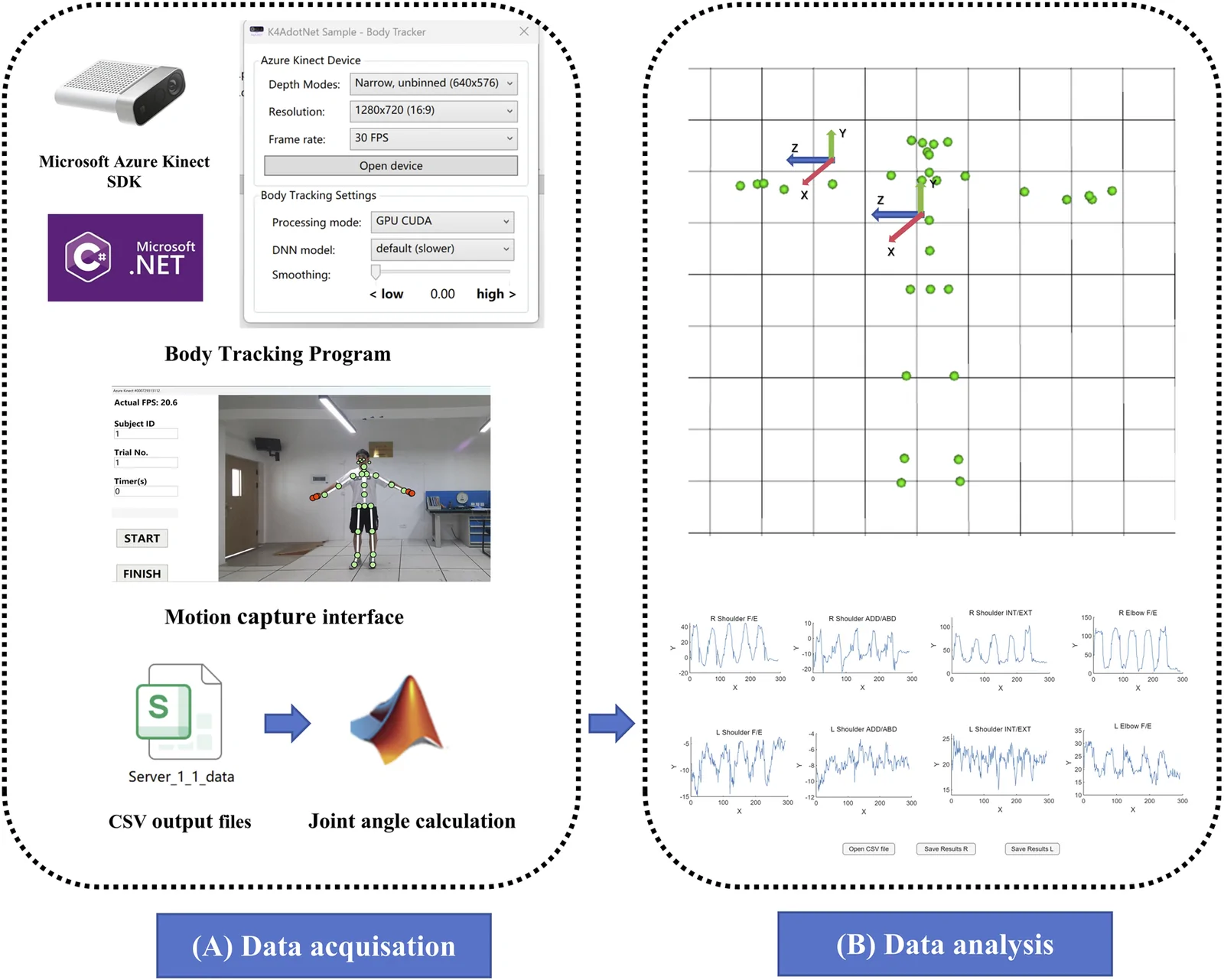
Overview of the Azure Kinect-based data acquisition and analysis pipeline. **(A)** The C#-based data acquisition interface showing real-time skeletal tracking and CSV export. **(B)** The Matlab-based post-processing pipeline for joint angle calculation from exported CSV data.

Participants were instructed to perform six functional tasks designed to simulate activities of daily living: axilla wash, hand-to-mouth movement, combing hair, perineal care, forward reach with a 1-kg load, and upward reach with a 1-kg load (Figure 3). These tasks were selected to mimic activities of daily life based on previous studies (Santago et al., 2017; van Andel et al., 2008; Vidt et al., 2016). In addition, three shoulder ROM tests were performed to evaluate shoulder flexion, abduction, and internal/external rotation. All participants performed the tasks with their affected side. These tasks were performed in a fixed sequence to reduce the time burden on this clinical population, with rest periods provided between tasks as needed. The 1-kg load used for forward and upward reach was standardized for all participants. Compensatory trunk movement was not constrained during task performance in order to capture natural movement patterns reflective of real-world function. Prior to formal testing, participants were allowed to perform practice trials to familiarize themselves with each task. Standardized verbal instructions and demonstrations were provided by the investigator. Each task was performed at a self-selected comfortable speed and repeated five times to ensure measurement reliability. Details of these tasks were described in Table 1.

**FIGURE 3:**
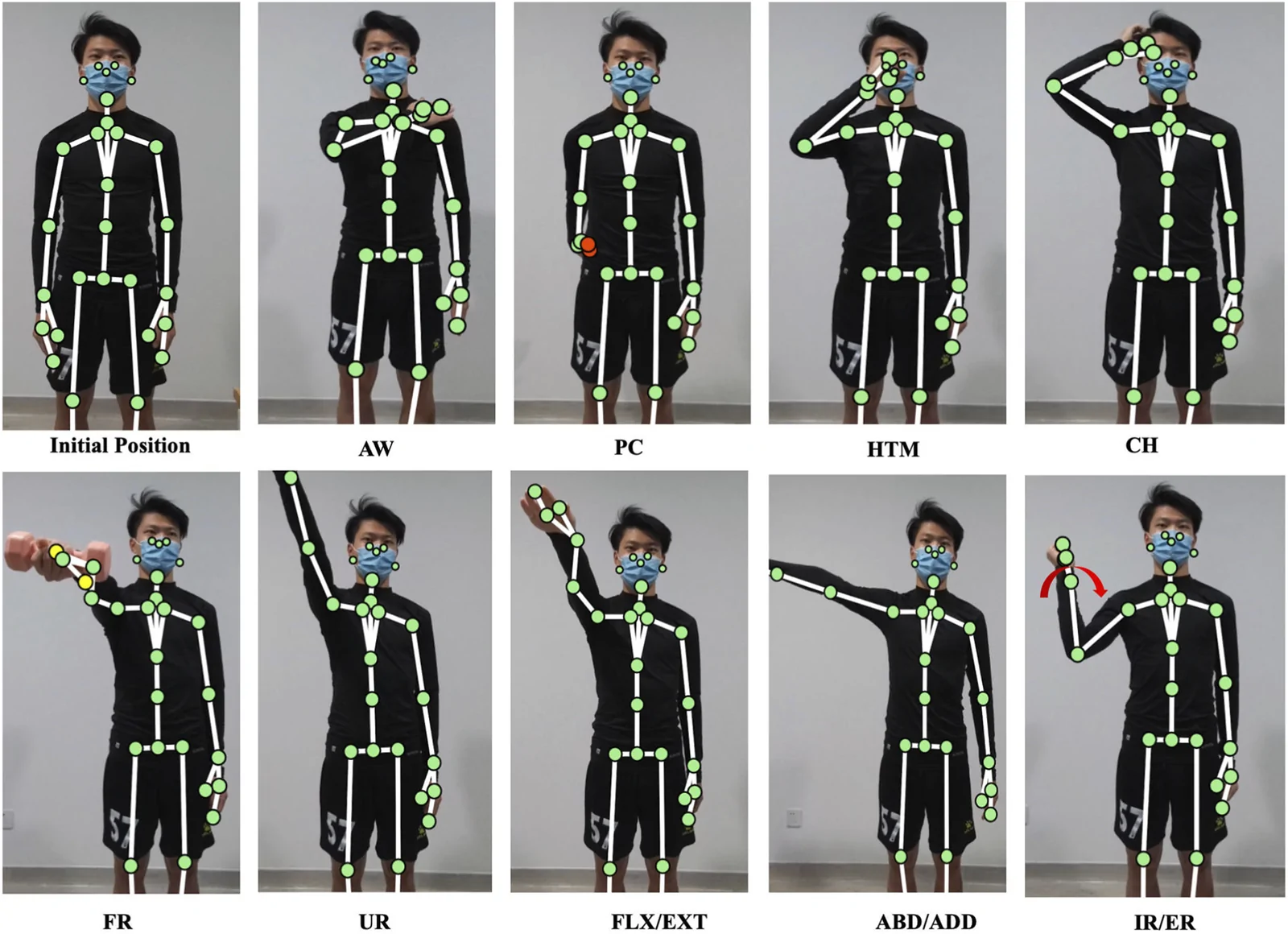
ADL-like task set and ROM test positions. The six functional tasks are illustrated: axilla wash (AW), hand-to-mouth (HTM), combing hair (CH), perineal care (PC), forward reach with a 1-kg load (FR), and upward reach with a 1-kg load (UR). Shoulder external rotation was performed with the shoulder abducted at 90° and the elbow flexed at 90°.

**TABLE 1:** Descriptions of selected upper limb functional tasks performed by patients with RCT. Patients returned their hands to the starting position after completing these tasks.

Task	Primary movement plane	Task instructions
Axilla wash (AW)	Frontal	Patients start with the arm in the anatomical and neutral position, and reached across the torso to touch the contralateral shoulder. This task mimics washing axilla or zipping up a jacket.
Hand to mouth (HTM)	Sagittal	Patients start with the arm in the anatomical and neutral position, and moved up to their mouth. This task mimics drinking water or eating.
Combing hair (CH)	Sagittal and frontal	Patients start with the arm in the anatomical and neutral position, and moved to their forehead and over their head to neck. This task mimics washing and combing hair.
Perineal care (PC)	Sagittal	Patients start with the arm in the anatomical and neutral position, and reached behind their torso and touch the middle of lumbar with the palm side. This task mimics perineal care.
Forward and upward reach with a 1 kg item (FR and UR)	Sagittal	Patients start with the elbow flexed to 90° and upper arm parallel to the torso, then upward and forward reach as far as possible with an 1 kg item. This task mimics taking an item from a cupboard and cleaning a desk.
3D shoulder range of motion test	Sagittal/Frontal/Transversal	Patients were asked to flex/extend, adduct/abduct and internally/externally rotate the shoulder at their best.

### 2.3 Data analysis

IMU-based 3D upper limb joint angles, including shoulder flexion/extension, adduction/abduction, internal/external and elbow flexion/extension joint kinematics were computed via the MR3 (MyoMotion, Noraxon Inc., Scottsdale, AZ, United States) embedded sensor fusion algorithms. Kalman filtering was applied to minimize drifting of the gyroscope or metallic disturbances of the magnetometer. According to ISB recommendations, 3D shoulder joint angles were calculated using planar angle algorithms to adjust rotation matrix to the major motion plane of a given activity. Elbow joint angle was locked to 1D. Axial rotation of the upper arm was corrected to match the orientation of the forearm so that the elbow angle was pure flexion.

Azure Kinect-based joint angles were calculated by a customized program in Matlab R2023b (MathWorks Inc., Natick, MA, United States). This program was well-documented and followed a similar kinematic modelling approach as previous studies using Kinect-based systems for upper limb motion analysis (Cai et al., 2021; Cai et al., 2019; Ma et al., 2020). The complete Matlab code is provided in the Supplementary Material to ensure reproducibility of the analysis.

All the body landmarks were filtered by a fourth-order Butterworth low-pass filter with a cut-off frequency of 6 Hz. Shoulder rotational joint angles were defined as Euler between the thoracic and humerus coordinate systems as recommended by the ISB (Wu et al., 2005). As shown in Figure 2B, Table 2 and Equation 1.

$$\text{TF}=\left[segement\;origin,Line1,Line\;2,definition\;order\right]$$ (1)

**TABLE 2:** Technical frames of humerus and thoracic coordinate system in the Azure Kinect-based motion capture system.

Segment	Segment origin	Line 1	Line 2	Definition order
Thoracic	Spine_Chest landmark	The vector from the Spine_Naval landmark to segment origin	The vector from the RShoulder to the LShoulder landmark	YXZ
Humerus	Elbow landmark	The vector from the elbow to the shoulder landmark	The vector from the wrist to the elbow landmark	YZX

For the thoracic segment, the origin was the middle spine landmark (“Spine_Naval”). Line 1 (y-axis) was the normalized vector from the “Spine_Naval” to the “Spine_Chest” landmark. Line 2 (x-axis) was the normalized vector from the right shoulder (“RShoulder”) to the left shoulder (“LShoulder”) landmark. The z-axis was derived from the cross product of x and z-axis in a YXZ order. For the humeral segment, the origin was the elbow (“RElbow or LElbow” depend on the affected side) landmark. Line 1 (y-axis) was the normalized vector from the elbow the ipsilateral shoulder landmark. Line 2 (z-axis) was the normalized vector from the elbow to the ipsilateral wrist (“RWrist or LWRist” depend on the affected side). The x-axis was derived from the cross product of y and z-axis in a YZX order. Figure 3 illustrates the thoracic and humeral segmental coordinate systems.

The relative orientation between the thoracic and humeral technical frames was expressed as the rotation matrix $R_{ZXY}\left(\alpha ,\beta ,\gamma \right)$ that rotated the ${TF}_{Thoracic}$ to the ${TF}_{\text{Humerus}}$ (Equation 2).

$${TF}_{\text{Humerus}}=R_{ZXY}\left(\alpha ,\beta ,\gamma \right)\times {TF}_{Thoracic}$$ (2)

Following a Z-Y-X rotation order (Equation 3), the Euler angles were decomposed using Equations 4–7, where α (z-axis) represents shoulder flexion/extension, β (y-axis) represents shoulder adduction/abduction angle, and γ (y-axis) represents shoulder internal/external rotation. For the left upper limb, the signs of the x-axis and y-axis were reversed to maintain consistent clinical interpretation, as recommended by the ISB.

$$R_{ZXY}\left(\alpha ,\beta ,\gamma \right)=\left[\begin{array}{ccc} -\sin\alpha \sin\beta \sin\gamma +\cos\alpha \cos\gamma & -\sin\alpha \cos\beta & \sin\alpha \sin\beta \cos\gamma +\cos\alpha \cos\gamma \\ \cos\alpha \sin\beta \sin\gamma +\sin\alpha \cos\gamma & \cos\alpha \cos\beta & -\cos\alpha \sin\beta \cos\gamma +\sin\alpha \sin\gamma \\ -\cos\beta \sin\gamma & \sin\beta & \cos\beta \cos\gamma \end{array}\right]$$ (3)

$$R_{ZXY}\left(\alpha ,\beta ,\gamma \right)=\left[\begin{array}{ccc} e_{11} & e_{12} & e_{13}\\ e_{21} & e_{22} & e_{23}\\ e_{31} & e_{32} & e_{33} \end{array}\right]$$ (4)

$$\beta =\mathrm{atan}\left(e_{32}/\sqrt{{e_{12}}^{2}+{e_{22}}^{2}}\right)$$ (5)

$$\alpha =\mathrm{atan}\left(-\frac{e_{12}}{e_{22}}\right)$$ (6)

$$\gamma =\mathrm{atan}\left(-\frac{e_{31}}{e_{33}}\right)$$ (7)

The upper arm vector was defined as the vector from the elbow to the wrist landmark, and the forearm vector was defined as the vector from the elbow to the shoulder landmark. The elbow flexion/extension angle was then calculated as the angular deviation from full extension, as shown in Equation 8. This formulation yields 180 ° when the arm is fully extended, consistent with clinical goniometric conventions.

$$\theta_{\text{Elbow}}=180{}^{\circ}-\cos^{-1}\left(\frac{\vec{v_{\text{Forearm}}}\cdot \vec{v_{\text{Upper}\;\text{arm}}}}{\left|\vec{v_{\text{Forearm}}}\right|\left|\vec{v_{\text{Upper}\;\text{arm}}}\right|}\right)\times \frac{180{}^{\circ}}{\pi \;}$$ (8)

It should be noted that the Azure Kinect-derived shoulder angles represents shoulder-complex motion rather isolated glenohumeral joint rotation, as the SDK defines humeral and thoracic coordinate systems using a simplified set of landmarks. This distinction is considered in the interpretation of our results.

For the six ADL tasks, based on previous studies demonstrating that Kinect-based motion capture systems achieve the best accuracy for elbow flexion/extension angle (Beshara et al., 2021; Cai et al., 2019), we used the elbow flexion/extension angle profiles as the reference for defining movement cycles for both the Azure Kinect and IMU systems. The event detection procedure first identified local maxima and minima in the elbow angle waveform to locate candidate movement events. These automatically detected events were then visually verified and manually adjusted if necessary to ensure accurate identification of trial onset and offset. These frames were applied to segment all other joint angles. For shoulder 3D range of motion tests, the onset and offset of each trial were identified independently from the peak and valley points in their respective angle trajectories. The semi-automated approach was applied consistently across all trials and participants. Only the steady-state movement phases were analyzed.

After the event detection procedure, the segmented movement cycles were temporally normalized to 101 data points using linear interpolation, with 0% representing the initial of hand movement and 100% representing the return of the hand to the starting position. For each participant, joint waveforms from five repeated trials were averaged for further statistical analysis.

### 2.4 Statistical analysis

ROM values were extracted from the test data during the data process. The normality of all ROM values was assessed using the Shapiro-Wilk test (p > 0.05). For parameters with a normal distribution, the paired-samples t-test was used; for parameters without a normal distribution, the Wilcoxon signed-rank test was used.

To assess the agreement between the two motion capture systems, the intra-class correlation coefficient (ICC), Lin’s concordance correlation coefficient (CCC), coefficient of multiple correlation (CMC), Pearson’s correlation coefficient (r), and Bland-Altman plot with standard error of measurement (SEM) were selected. Bland-Altman agreement analyses were supplemented with regression of the differences against the means to assess proportional bias. Measurement differences of joint angle waveform generated by the two systems were evaluated by root mean square error (RMSE) and mean absolute error (MAE). ICC and their 95% confidence intervals were calculated using SPSS statistical package version 27.0 (SPSS Inc, Chicago, IL, United States) based on a single rating and a mean rating (k = 5), absolute-agreement, two-way random-effects model (Koo and Li, 2016), denoted as ICC_2,5_. The standard error of measurement (SEM) was calculated as an indicator of measurement variability using the formula SEM = SD × √(1 − ICC), where ICC was derived from the absolute-agreement two-way random-effects model (ICC_2,5_). The SD represented the pooled standard deviation of measurements obtained from both systems across participants.

As noted by Koo and Li (2016), ICC values below 0.5 indicate poor agreement, values between 0.5 and 0.75 indicate moderate agreement, values between 0.75 and 0.9 indicate good agreement; and values above 0.90 indicate excellent agreement. The limits of agreement (LOAs) were expected to include 95% of the differences between measurement systems and were defined as mean ±1.96 × standard deviations.

## 3 Results

Figure 4 illustrates the joint angle waveforms of selected upper limb functional tasks, depicting average segment rotation angles captured by the Azure Kinect and IMU systems across five testing sessions.

**FIGURE 4:**
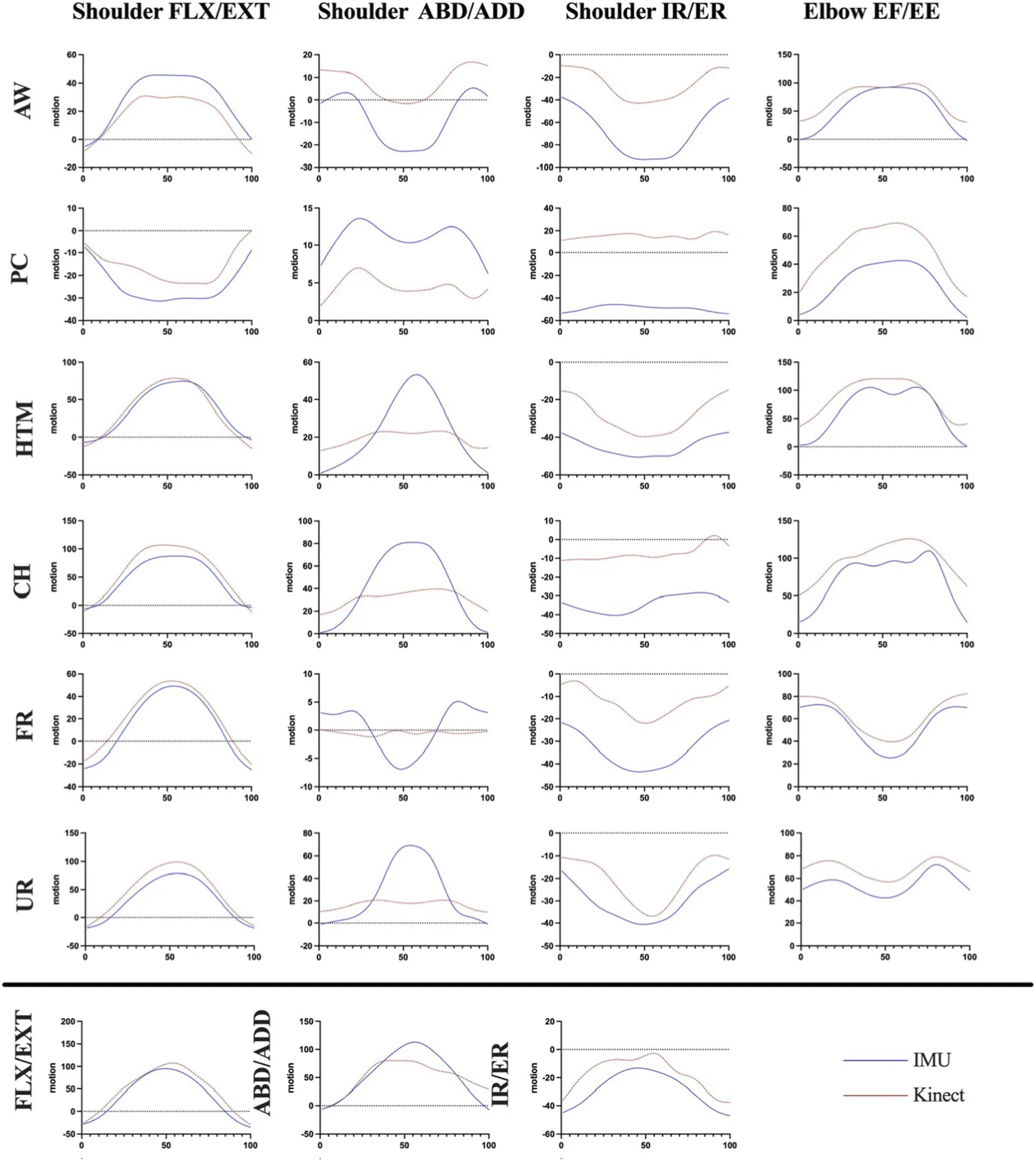
Time-normalized joint angle waveforms (0%-100% of the movement cycle) obtained from the Azure Kinect and IMU systems for selected tasks. Curves represent group mean across five repetitions per task (n=12) for shoulder flexion/extension (FL/EXT), abduction/adduction (ABD/ADD), internal/external rotation (IR/ER), and elbow flexion/extension (EF/EE).

### 3.1 Validity of Azure Kinect for upper limb functional assessment in RCT patients

As shown in Table 3, shoulder flexion angles demonstrated good to excellent agreement (ICC_2,5_ > 0.75, r > 0.75) in the PC (ICC_2,5_ = 0.751, r = 0.802), HTM (ICC_2,5_ = 0.907, r = 0.923), and CH (ICC_2,5_ = 0.889, r = 0.893) tasks. The HTM task showed the best inter-system agreement, while moderate agreement (0.50 < ICC_2,5_ < 0.75) was observed in the AW (ICC_2,5_ = 0.600, r = 0.607), FR (ICC_2,5_ = 0.708, r = 0.712), and UR (ICC_2,5_ = 0.733, r = 0.748) tasks. CMC and CCC values indicated good to excellent waveform consistency (CMC > 0.75; CCC > 0.70) for the HTM, CH, FR, and UR tasks, with relatively higher RMSE and MAE observed in the PC and CH tasks compared with the others.

**TABLE 3:** Inter-system agreement between Azure Kinect and IMU for four joint angles during six functional tasks simulating activities of daily life. Values are CMC, RMSE (°), MAE (°), CCC, ICC_2,5_ (two-way random-effects, absolute-agreement; mean of five repetitions), and Pearson’s r with their 95% CI.

Joint angle	Metrics	AW	PC	HTM	CH	FR	UR
Shoulder FLX/EX	ICC (95% CI)	0.600 (−0.004 to 0.883)	0.751 (0.309–0.926)	0.907 (0.672–0.976)	0.889 (0.618–0.971)	0.708 (0.223–0.912)	0.733 (0.235–0.926)
	r	0.607 (−0.037,0.895)	0.802 (0.389,0.947)	0.923 (0.701,0.982)	0.893 (0.600,0.975)	0.712 (0.196,0.919)	0.748 (0.225,0.937)
	CMC	0.687 ± 0.294	0.319 ± 0.443	0.898 ± 0.715	0.862 ± 0.101	0.899 ± 0.082	0.903 ± 0.082
	RMSE (°)	16.8 ± 9.7	25.3 ± 15.0	18.5 ± 6.1	27.6 ± 13.5	16.0 ± 6.6	21.1 ± 10.4
	MAE (°)	15.2 ± 9.7	24.0 ± 15.1	15.6 ± 5.7	23.6 ± 12.0	14.8 ± 6.7	18.8 ± 9.6
	CCC (95% CI)	0.611 (0.503,0.718)	0.330 (0.196,0.464)	0.826 (0.785,0.866)	0.776 (0.719,0.832)	0.834 (0.791,0.877)	0.839 (0.790,0.887)
Shoulder ABD/ADD	ICC (95% CI)	0.694 (0.157–0.914)	0.688 (0.185-0.905)	0.226 (−0.435 to 0.729)	0.053 (−0.567 to 0.635)	0.612 (0.055–0.878)	0.125 (−0.516 to 0.676)
	r	0.859 (0.500,0.966)	0.690 (0.153,0.912)	0.665 (0.062,0.983)	0.114 (-0.556,0.694)	0.612 (0.019,0.886)	0.621 (-0.014,0.899)
	CMC	0.078 ± 0.127	0.188 ± 0.325	0.331 ± 0.313	0.314 ± 0.340	0.043 ± 0.141	0.326 ± 0.320
	RMSE (°)	19.1 ± 6.6	15.1 ± 11.5	23.2 ± 12.6	33.8 ± 19.8	20.4 ± 5.4	33.4 ± 24.4
	MAE (°)	17.8 ± 6.0	14.4 ± 11.6	19.8 ± 9.4	28.3 ± 15.2	19.3 ± 5.9	28.3 ± 19.0
	CCC (95% CI)	0.208 (0.144,0.271)	0.134 (0.017,0.251)	0.240 (0.157,0.323)	0.263 (0.179,0.347)	0.143 (0.088,0.197)	0.232 (0.135,0.329)
Shoulder IR/ER	ICC (95% CI)	0.618 (0.025–0.889)	0.286 (−0.347 to 0.740)	0.021 (−0.588 to 0.615)	0.525 (−0.113 to 0.856)	−0.236 (−0.715 to 0.393)	0.140 (−0.540 to 0.709)
	r	0.620 (−0.016,0.899)	0.462 (−0.191,0.831)	0.023 (−0.615,0.614)	0.562 (−0.104,0.880)	−0.245 (−0.736,0.416)	0.182 (−0.549,0.755)
	CMC	0.408 ± 0.359	0.056 ± 0.187	0.182 ± 0.318	0.337 ± 0.329	0.039 ± 0.130	0.332 ± 0.362
	RMSE (°)	48.0 ± 37.1	67.3 ± 26.1	21.9 ± 14.8	34.6 ± 36.0	23.6 ± 10.3	23.2 ± 18.8
	MAE (°)	45.5 ± 37.0	65.6 ± 27.5	20.0 ± 15.3	32.9 ± 36.0	22.3 ± 10.4	21.1 ± 18.4
	CCC (95% CI)	0.378 (0.252,0.504)	0.013 (−0.038,0.065)	0.205 (0.108,0.301)	0.242 (0.128,0.356)	0.138 (0.115,0.160)	0.294 (0.182,0.406)
Elbow FLX/EX	ICC (95% CI)	0.388 (−0.280 to 0.802)	0.484 (−0.127 to 0.829)	0.050 (−0.601 to 0.661)	0.212 (−0.485 to 0.744)	0.846 (0.527–0.956)	0.804 (0.390–0.947)
	r	0.474 (−0.222,0.850)	0.489 (−0.157,0.842)	0.055 (−0.535,0.709)	0.213 (−0.525,0.769)	0.861 (0.541,0.963)	0.805 (0.356,0.952)
	CMC	0.665 ± 0.267	0.208 ± 0.364	0.776 ± 0.130	0.570 ± 0.353	0.711 ± 0.372	0.536 ± 0.393
	RMSE (°)	32.4 ± 9.7	45.5 ± 25.3	34.5 ± 12.5	42.0 ± 27.0	15.4 ± 8.7	19.8 ± 11.3
	MAE (°)	29.1 ± 7.6	41.6 ± 25.0	29.9 ± 10.0	33.8 ± 20.9	14.1 ± 8.7	17.6 ± 11.2
	CCC (95% CI)	0.541 (0.438,0.645)	0.233 (0.140,0.325)	0.657 (0.589,0.725)	0.502 (0.386,0.617)	0.705 (0.603,0.808)	0.585 (0.484,0.685)

Abbreviations: FLX/EXT, flexion/extension; ABD/ADD, abduction/adduction; IR/ER, internal/external rotation; EF/EE, elbow flexion/extension; AW, axilla wash; PC, perineal care; HTM, hand-to-mouth; CH, comb hair; FR, forward reach with 1-kg load; UR, upward reach with 1-kg load.

Shoulder abduction angles achieved moderate agreement in the AW (ICC_2,5_ = 0.694, r = 0.859), PC (ICC_2,5_ = 0.688, r = 0.690), and FR (ICC_2,5_ = 0.612, r = 0.612) tasks, with lower agreement (ICC_2,5_ < 0.50) in the HTM (ICC_2,5_ = 0.226, r = 0.665), UR (ICC_2,5_ = 0.125, r = 0.621) and CH (ICC_2,5_ = 0.053, r = 0.114) tasks. CMC and CCC values were consistently low across all tasks, indicating poor to moderate waveform consistency between the two systems. Larger RMSE and MAE values were observed in the CH and UR tasks, reflecting greater measurement discrepancies in these movements.

Shoulder horizonal rotation angles showed moderate agreement in the AW and CH tasks (ICC_2,5_ = 0.618 and 0.525, respectively), with poor agreement in the PC, UR, FR and HTM tasks (ICC_2,5_ < 0.50). CMC and CCC values were consistently low across all tasks, suggesting limited waveform similarity. RMSE and MAE values were substantially larger for shoulder horizonal rotation compared with the other joint degrees of freedom, indicating the greatest measurement error in this movement plane.

Elbow flexion angles demonstrated high agreement in the FR and UR tasks (ICC_2,5_ = 0.846 and 0.804, respectively), moderate agreement in the PC task (ICC_2,5_ = 0.484), and lower agreement (ICC_2,5_ < 0.50) in the AW, HTM, and CH tasks. CMC and CCC values indicated poor to moderate waveform consistency for most tasks, with the exception of the HTM task, where better consistency was observed (CMC = 0.776 ± 0.130). Smaller RMSE and MAE values were found in the UR and FR tasks.

The measurement consistency between the two systems was evaluated using the Bland-Altman agreement analysis method. The bias-mean scatter plots are presented in Figure 5, with the mean measurement bias and 95% LOA detailed in Table 4. The results indicated that measurement data from both systems exhibited a random distribution around the zero-bias line, with the majority of values falling within the LOA intervals. The Azure Kinect demonstrated high consistency in shoulder flexion, with a narrow LOA range and most SEM <15°. However, it showed significant errors in coronal plane abduction (e.g., in the CH and UR tasks) and horizontal plane rotation (e.g., in the PC task). Regression analyses of the differences against the means revealed significant proportional bias for most joint planes and tasks (p < 0.05), indicating that the measurement error varied with the magnitude of the measured angle.

**FIGURE 5:**
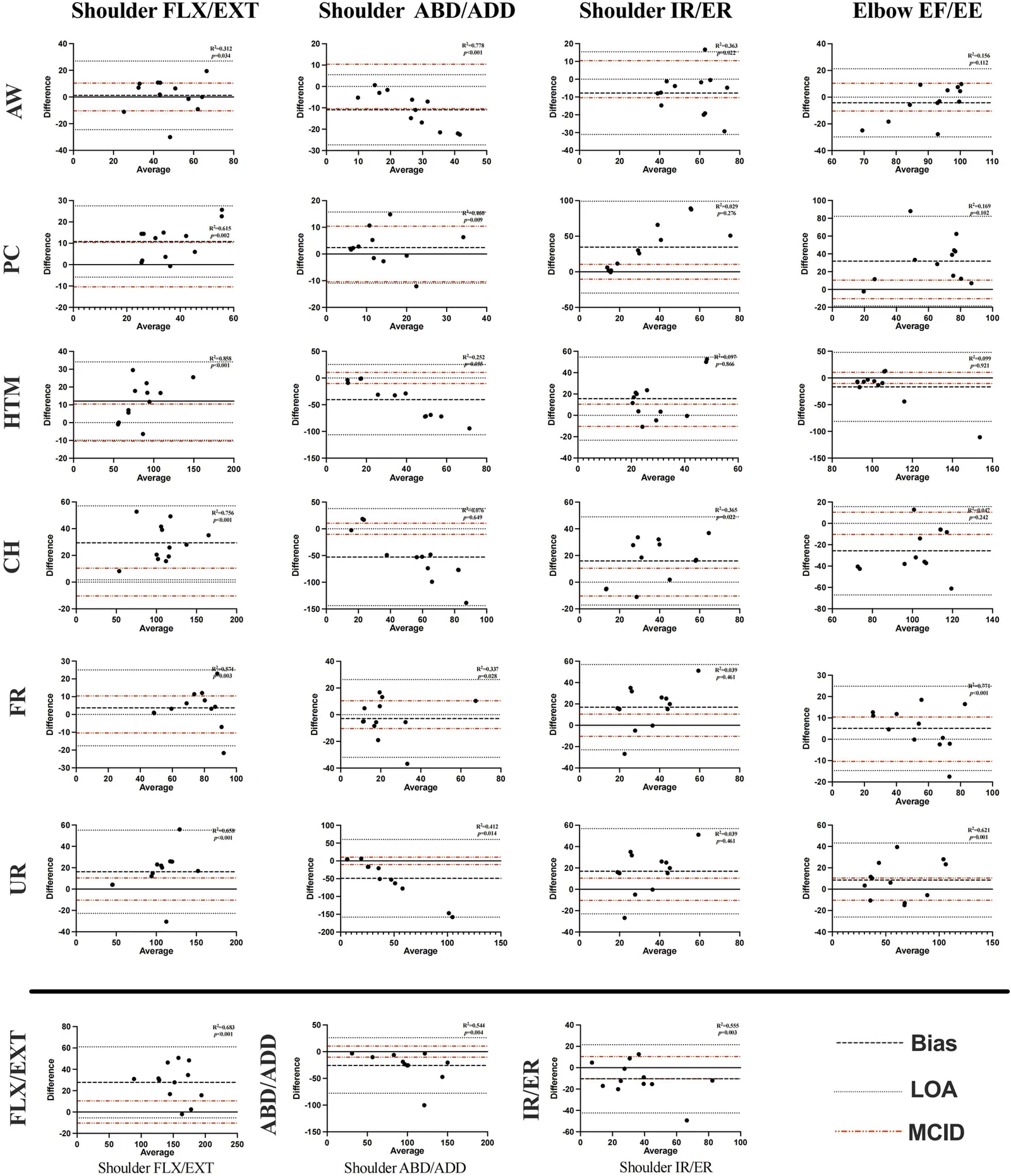
Bland–Altman plots comparing joint angles measured by the Azure Kinect and IMU systems across the six tasks. The solid line represents the mean bias (Kinect–IMU), and the dashed lines represent the 95% limits of agreement (LOA). A positive bias indicates that the Azure Kinect overestimates joint angles relative to the IMU. The red dashed lines represent the MCID values.

**TABLE 4:** Bland–Altman agreement between Azure Kinect and IMU for joint angles across six functional tasks simulating activities of daily life. For each joint/plane, values are Bias (Azure Kinect–IMU, °), 95% limits of agreement (LOA, lower–upper, °), standard error of measurement (SEM, °). Positive bias indicates larger angles measured by Azure Kinect.

Task	Shoulder FLX/EXT			Shoulder ABD/ADD			Shoulder IR/ER			Elbow FLX/EXT		
​	Bias	LOA	SEM	Bias	LOA	SEM	Bias	LOA	SEM	Bias	LOA	SEM
AW	1.2	−23.5 to 26.9	9.2	−10.9	−27.3 to 5.5	6.7	−7.8	−31.1 to 15.4	8.9	−4.2	−29.8 to 21.3	9.1
PC	10.8	−5.9 to 27.5	7.0	2.4	−10.9 to 15.7	5.1	34.6	−30.0 to 99.1	28.4	31.8	−18.6 to 82.2	22.4
HTM	12.1	−9.9 to 34.1	7.7	−40.5	−106.2 to 25.2	30.1	15.6	−23.3 to 54.5	21.1	−16.8	−81.4 to 47.9	29.1
CH	29.4	1.72–57.1	10.6	−52.9	−143.7 to 37.9	41.6	15.9	−17.2 to 48.9	13.9	−25.7	−67.1 to 15.7	19.9
FR	3.7	−17.6 to 25.0	9.0	−2.8	−32.0 to 26.3	8.3	−1.3	−38.4 to 35.8	14.8	5.1	−14.7 to 24.8	7.9
UR	16.2	−22.8 to 55.2	18.4	−49.0	−158.1 to 60.1	49.2	16.9	−23.0 to 56.9	20.0	8.5	−26.1 to 43.2	11.6

### 3.2 Validity of Azure Kinect for shoulder ROM evaluation in RCT patients pre- and post-surgery

As shown in Table 5, the Azure Kinect demonstrated high levels of agreement in ROM measurement tasks, including shoulder flexion (ICC_2,5_ = 0.837), abduction (ICC_2,5_ = 0.729), and internal/external rotation (ICC_2,5_ = 0.735).

**TABLE 5:** Shoulder range of motion (ROM, °) measured preoperatively (ROM-pre) and at 4 months postoperatively (ROM-post) by Azure Kinect and IMU.

Metrics		FLX/EXT			ABD/ADD			IR/ER	
​	Kinect	IMU	*p*_*1*_	Kinect	IMU	*p*_*1*_	Kinect	IMU	*p*_*1*_
ROM-pre	165.9 ± 29.1	138.1 ± 33.1	0.061	87.0 ± 33.2	112.8 ± 45.5	0.151	32.0 ± 25.0	42.4 ± 33.0	0.848
ROM-post	137.9 ± 13.5	97.1 ± 25.1	0.002	94.3 ± 24.4	85.0 ± 31.5	0.480	48.6 ± 14.6	31.0 ± 14.4	0.038
*p*_*2*_	0.026	0.016	​	0.624	0.205	​	0.140	0.654	​
ICC_2,5_ (95% CI)	0.837 (0.504–0.953)			0.729 (0.225–0.925)			0.735 (0.059–0.949)		
r	0.844			0.765			0.768		
Bias	27.8			−25.86			−10.46		
LOA	−5.5 to 61.0			−77.8 to 26.1			−42.4 to 21.5		
SEM	8.1			14.7			7.5		

Abbreviations: *p*_*1*_: paired Kinect–IMU, at same time; p_2_: pre–post change within system.

In the measurement of shoulder flexion angle, preoperative results from Azure Kinect and IMU were 165.9° ± 29.1° vs. 138.1° ± 33.1° (p = 0.061), with no statistically significant difference between groups. Postoperative Kinect measurements were significantly higher than IMU (p = 0.002).

In the measurement of shoulder abduction angle, preoperative results from Azure Kinect and IMU were 87.0° ± 33.2° vs. 112.8° ± 45.5° (p = 0.151). Postoperative results were 94.3° ± 24.4° vs. 85.0° ± 31.5° (p = 0.480), both showing no statistically significant differences.

In the measurement of shoulder external rotation angle, preoperative results from Azure Kinect and IMU were 32.0° ± 25.0° vs. 42.4° ± 33.0° (p = 0.848), with no statistically significant difference between groups. Postoperative Kinect measurements were significantly higher than IMU (p = 0.038).

Both the Azure Kinect and IMU systems detected statistically significant differences in preoperative and postoperative flexion angles (p = 0.026 and p = 0.016, respectively). However, neither of the two systems was able to detect preoperative-to-postoperative changes in frontal and transverse planes (p > 0.05).

Overall, these findings should be interpreted with caution given the limited sample size, as will be further discussed in the following section.

## 4 Discussion

This study is the first to systematically evaluate the concurrent validity of the Azure Kinect for assessing upper limb kinematics in patients with RCTs and to examine its ability to detect postoperative shoulder ROM changes following arthroscopic repair. The results revealed that measurement validity was task- and plane-dependent. In multi-planar functional movement scenarios (i.e., AW, HTM), the Azure Kinect demonstrated higher measurement accuracy for sagittal plane kinematics, whereas measurement agreement for coronal and transverse joint angles was relatively lower. For single-plane basic motions (i.e., FL, ABD), Azure Kinect showed good measurement agreement with its IMU-based counterpart. Based on these preliminary results, the Azure Kinect appeared suitable as a screening tool for tracking gross functional recovery, but its concurrent validity was insufficient for use as a standalone quantitative clinical assessment tool, particularly due to its tendency to overestimate postoperative flexion and external rotation. However, given the small sample size, these findings should be interpreted with caution and require confirmation in larger cohorts.

The plane-dependent validity pattern was consistently reflected across multiple agreement metrics, including CCC, ICC_2,5_ and r. The Azure Kinect demonstrated high measurement consistency for shoulder flexion angles in multi-planar functional tasks. In sagittal-plane dominant FR and UR tasks, both shoulder flexion angles and elbow flexion angles showed substantial consistency. However, in movements involving coronal and transverse planes, the consistency of shoulder abduction, internal/external rotation, and elbow flexion angles was relatively low. This finding aligns with previous reports that single depth-camera systems capture large-amplitude sagittal-plane movements more accurately than multi-planar coupled motions or low-amplitude rotations (Huber et al., 2015; Jo et al., 2022; Ma et al., 2019). Potential mechanisms include that Kinect’s skeletal tracking relies on a global optimization model; limb occlusion during complex movements markedly reduces the accuracy of anatomical landmark recognition, and that small-amplitude motions such as shoulder external rotation, typically less than 60°, are susceptible to noise-induced trajectory distortion, whereas IMUs compute angular changes directly from inertial data and thus avoid visual-occlusion errors (Beshara et al., 2021).

Compared with IMU systems, Kinect shows distinct error behavior in multi-planar tasks. IMUs resolve motion by time-synchronizing accelerometer and gyroscope signals and applying filter algorithms, whereas Kinect estimates pose from RGB-D imagery with a skeletal model. During rapid, coordinated movements, self-occlusion or transient depth loss degrades landmark detection and inflates joint-position error (Proffitt et al., 2023; Sabatini, 2011). In addition, IMUs commonly use Kalman-type filtering to compensate drift and movement artifacts in real time, while Kinect updates a global reference through visual feature matching, which can accumulate registration error when motions span multiple planes, such as shoulder external rotation with concurrent trunk lateral flexion (Albert et al., 2020; Büker et al., 2023; Rammer et al., 2018). A hybrid motion capture protocol integrating Kinect and IMUs could reduce joint position tracking errors by up to 60% compared with Kinect alone, suggesting that such systems may be better suited for applications requiring higher precision, such as preoperative decision-making or subtle kinematic analysis, albeit at the cost of increased procedure complexity (Abbasi et al., 2021). It also should be noted that some planes and tasks (e.g., shoulder frontal angles in the HTM and UR tasks) exhibited low ICC but moderate-to-high Pearson’s r explicitly confirms that association and agreement are not equivalent in statistical terms. Specifically, while the high Pearson’s r indicates strong synchronization in movement trends and relative angular changes between the systems, the low ICC suggests the presence of systematic absolute offsets, implying that although both methods are reliable for tracking kinematic changes, their absolute joint angle measurements are not interchangeable.

For preoperative single-plane joint angle measurement tasks, the Azure Kinect demonstrated high consistency in measuring shoulder flexion, abduction, and internal/external rotation angles, which is related to the single-plane task design that requires subjects to perform movements with the maximum active ROM within that plane. This result is consistent with a previous study that tested the reliability of the Kinect V2 using marker-based systems, indicating that the magnitude of error was proportional to the range of joint angles (Cai et al., 2019). Thus, the measurement error of Azure Kinect is smaller in movements with a wider ROM and larger in movements with relatively restricted ROM (Cai et al., 2019; Çubukçu et al., 2020). To obtain more accurate measurements of shoulder internal/external rotation angles, subjects were instructed to perform external rotation with shoulders and elbows flexed at 90°, maximizing motion capture in the coronal plane, which is a method commonly used in clinical visual assessment (Cai et al., 2021; Seo et al., 2016).

Notably, although the ICC_2,5_ values suggested moderate agreement between the two systems for ROM measurements and no statistical significance was found, the mean difference between Azure Kinect and the IMU reference exceeded the established minimal clinically important difference (MCID = 10.4°) for shoulder outcomes (Kukkonen et al., 2013). These MCID-exceeding errors indicate that Azure Kinect is unreliable for preoperative functional assessment, as it may mask clinically meaningful improvements or changes in patients’ conditions. However, several considerations warrant a nuanced interpretation of this finding. First, given the wide confidence intervals observed for some ICC_2,5_ estimates, this finding should be considered preliminary and warrants further validation in larger samples. Second, the MCID was derived from the Constant score, which was a composite functional measure, rather than a pure ROM threshold; therefore, its direct application as a benchmark for ROM measurement error should be considered approximate. Current validation studies on Kinect-based shoulder motion measurement have predominantly focused on healthy populations and relied on ICC or coefficient of multiple correlation (Cai et al., 2019; Çubukçu et al., 2020). However, these statistical indices are insufficient for clinical validation because they measure relative agreement rather than absolute error, and are highly influenced by sample heterogeneity. This limitation is particularly relevant for postoperative rehabilitation populations, who exhibit significant movement variability and require greater measurement precision (Dabija and Jain, 2019; Faity et al., 2022). Therefore, we suggest that future validation studies for clinical movement analysis should explicitly compare measurement errors against established MCID thresholds to facilitate clinically meaningful interpretation (Dabija and Jain, 2019).

Alterations in shoulder ROM in patients with RCT are critical to evaluate arthroscopic repair outcomes (Antonacci et al., 2025). The postoperative assessment at 4 months revealed that Azure Kinect significantly overestimated both flexion and external rotation compared with IMUs. This overestimation may be partly attributed to the inherent limitations of the Azure Kinect-based biomechanical model described in the Methodology section. The SDK provides no additional upper arm landmarks beyond the shoulder and elbow. The wrist landmark, which anatomically belongs to the forearm, had to be used to define the humeral coordinate system, as three non-collinear points are the minimum required to construct a segmental coordinate system. Consequently, motions occurring at the forearm may become confounded with humeral kinematic assessment. The thoracic coordinate system was defined using the middle spine, chest and bilateral shoulder landmarks. Established evidence has shown that the shoulder complex and the trunk are adjacent links in a functional continuum, where inefficiency at the trunk level directly alters force transfer to the shoulder (Richardson et al., 2020). Yamamoto et al. (2015) found that postural abnormality, including kyphotic-lordotic, flat-back, and sway-back postures, represents an independent predictor for patients with rotator cuff tears. The relationship between trunk function and shoulder pathology is further supported by Sarıçimen et al. (2026), who found that individuals with rotator cuff disorders exhibit significantly lower abdominal muscle strength and endurance compared with healthy controls. These findings suggest that rotator cuff pathology is associated with broader impairments within the kinetic chain, including trunk musculature and posture, which may contribute to altered trunk movement patterns during arm elevation. Therefore, kinetic chain exercises that incorporate hip and trunk movement are clinically recommended (Yamauchi et al., 2015).

While the following mechanism is biomechanically plausible, it remains speculative in the absence of direct trunk kinematic data from our study. In the context of the Kinect-based assessment, these trunk motion alterations may affect the shoulder angle estimation. Because Kinect defines the shoulder angle as the relative orientation between the arm and thoracic coordinate system that is itself derived from trunk landmarks (the middle spine landmark), the system cannot decouple trunk contributions from shoulder-complex motion. As a result, if patients adopt compensatory trunk strategies, these trunk movements may alter the thoracic coordinate system, and could be attributed to shoulder angle calculation, potentially contributing to the overestimation observed postoperatively. To our knowledge, no study has quantified trunk compensation specifically for patients undergoing arthroscopic rotator cuff repair. Future studies incorporating simultaneous motion analysis of the trunk and shoulder are needed to further validate this proposed mechanism.

Despite these limitations, the Azure Kinect retains some clinical utility as a screening and adjunctive tool for postoperative functional tracking. A recent narrative review has highlighted that markerless motion capture, including depth sensors, offers a promising alternative for clinical shoulder ROM assessment, being user-friendly, time-efficient, and objective (van den Hoorn et al., 2025). Our results demonstrate that the Azure Kinect captured a significant postoperative decrease in shoulder sagittal ROM and no significant changes in shoulder frontal and transversal ROM, a trend consistent with IMU-based assessments. Such trend-level agreement has clinical values in scenarios where clinical decisions do not require precise absolute angle measurements. For example, in screening or triage settings, the Azure Kinect is able to distinguish patients with shoulder pathology from healthy individuals, as demonstrated in adhesive capsulitis (Lee et al., 2015). The Azure Kinect’s consistency with IMU also supports its utility for serial, low-cost gross function surveillance. Supplementary analyses of inter-repetition reliability further revealed that the Azure Kinect demonstrated levels of relative reliability comparable to those of the IMU reference system, as detailed in the Supplementary Material (Supplementary Table S1). However, formal test-retest and inter-session reliability studies are still needed to confirm this observation.

The Azure Kinect-powered Monitored Augmented Rehabilitation System has been validated as a reliable and accurate alternative for clinical shoulder and elbow ROM assessment, supporting its potential integration into telerehabilitation platforms for remote progress monitoring (Lauman et al., 2025). Beyond movement assessment, the Azure Kinect’s data-driven motion capture capability has the potential to be integrated with motor learning and rehabilitation principles to deliver personalized, feedback guided training for patients with neuromusculoskeletal disorders (Jiao et al., 2025). Given the relatively high accuracy of the Azure Kinect for sagittal shoulder and elbow angles, the data derived from this system may serve as a feasible tool for investigating shoulder-elbow inter-joint coordination during functional tasks (Dubois et al., 2023). In contrast to gait analysis where errors exceeding 5°, may lead to misleading interpretations (McGinley et al., 2009), no universally accepted measurement error threshold for shoulder kinematic assessment has been established. Thakkar et al. (2026) suggested that a RMSE range of 5°–10° was tolerable for clinical applications in IMU-based shoulder kinematic assessment. Measurement errors observed in this study substantially exceed these reported thresholds. Therefore, the Azure Kinect in its current form should not be used as a standalone quantitative instrument for high-stakes clinical decision-making, such as determining the indication for surgical intervention.

This study has several limitations. First, the sample size (n = 12) is small that may affect the precision of correlation coefficient estimation and statistical power, and restricts generalizability to patients with different tear severities, pathologies, and surgical procedures. This study was designed as an exploratory investigation, and the findings should therefore be viewed as preliminary rather than definitive. The wide confidence intervals observed for several ICC_2,5_ estimates underscore the uncertainty in our agreement metrics and highlight the need for larger-scale, multi-center studies with repeated measurement sessions and independent cohorts to validate our findings before any clinical implementation can be recommended. A healthy control group should also be recruited to further assess the discriminant validity of the Azure Kinect. Second, the task design was laboratory-based and did not include real-world activities of daily living scenarios (e.g., dressing, lifting). Pain was not systematically recorded during task performance. We cannot rule out the possibility that pain levels influenced movement patterns and contributed to between-subject variability. Third, neither the inter-session nor inter-day reliability was evaluated, hindering a comprehensive evaluation of the Azure Kinect as a clinical shoulder assessment tool. Fourth, the effect of clothing or body habitus on skeletal tracking accuracy was not examined. Fifth, although an IMU system served as the reference comparator, it has inherent limitations. IMU measurements are affected by the sensor placement protocol, calibration posture, potential drift, soft tissue artifact, and sensor-to-segment alignment errors. The Noraxon MyoMotion IMU system shows larger measurement errors in shoulder frontal and transverse planes than in the sagittal-plane (Bartoszek et al., 2022; Yoon, 2017). This plane-dependent error pattern suggests that some of the discrepancies observed in non-sagittal movements may reflect IMU inherent limitations rather than Azure Kinect tracking inaccuracies. Tolerate accuracy of IMUs has been investigated when tracking shoulder kinematics (Li et al., 2024; Morrow et al., 2017; Thakkar et al., 2026). Future studies should therefore consider employing gold standard optoelectronic marker-based motion capture systems as the reference to further validate the findings established in the present study, particularly for the frontal and transversal movements where the limitations of IMUs are most pronounced. Finally, the validity of Azure Kinect for detecting longitudinal changes at later postoperative time points, for example, when recovery is more complete, remains to be established and should be prioritized in future research.

## 5 Conclusion

In this exploratory study, the Azure Kinect exhibits task-dependent validity in multi-planar motion assessment for patients with RCT, showing superior measurement validity in the sagittal plane compared with coronal and transverse planes. Although the device demonstrates good agreement in single-plane measurements of shoulder flexion, abduction, and external rotation, it significantly overestimates shoulder flexion and external rotation angles when evaluating preoperative-to-postoperative ROM changes. These preliminary findings suggest that the Azure Kinect is not yet sufficiently accurate as a standalone quantitative tool for surgical outcome assessment. However, given the small sample size and the exploratory nature of this study, large-scale investigations are therefore needed to confirm these findings and inform clinical translation.

## Data Availability

The raw data supporting the conclusions of this article will be made available by the authors, without undue reservation.
