# Supplementary material for: Validation of Azure Kinect for upper limb kinematic assessment after arthroscopic rotator cuff repair: task-dependent accuracy and postoperative overestimation
Source: Front Bioeng Biotechnol. 2026 Sep 22;14:1927386. doi: 10.3389/fbioe.2026.1927386 (PMC13638575; doi:10.3389/fbioe.2026.1927386)
Supplement: Supplementary file 1 [file Table1.docx]

Supplementary Material

1. Matlab code for Azure Kinect-based upper limb joint angle calculation

[filename,pathname]=uigetfile('*.CSV','Select your data file');

if filename==0

return

end

str=fullfile(pathname,filename);

SkeletonWhole=csvread(str,2,2);

MiddleSpine1=SkeletonWhole(:,4:6);

MiddleSpine=filtfilt(b,a,MiddleSpine1);

SpineChest1=SkeletonWhole(:,7:9);

SpineChest=filtfilt(b,a,SpineChest1);

ShoulderR=SkeletonWhole(:,37:39);

ShoulderL=SkeletonWhole(:,16:18);

ElbowR=SkeletonWhole(:,40:42);

ElbowL=SkeletonWhole(:,19:21);

WristR=SkeletonWhole(:,43:45);

WristL=SkeletonWhole(:,22:24);

%Define thoracic coordinate system

token = 'yxz';

originTHO=SpineChest;

segmentOrigin = originTHO;

[originTHO, xTHO, yTHO, zTHO] = ...

defineSegment(segmentOrigin,SpineChest-MiddleSpine,ShoulderL-ShoulderR,token);

TOR = [xTHO,yTHO,zTHO];

%Define right upper arm coordinate system

token = 'yzx';

originRUA=ElbowR;

segmentOrigin = ElbowR;

[originRUA, xRUA, yRUA, zRUA] = ...

defineSegment(segmentOrigin,ShoulderR-ElbowR,WristR-ElbowR,token);

RUA = [xRUA,yRUA,zRUA];

for i=1:length(xRUA)

r=reshape(THO(i,:), 3, 3)'* reshape(RUA(i,:), 3, 3); rsa(i)=atan2(r(3,2),(r(2,2)^2+r(1,2)^2)^0.5)*180/pi;

rsb(i)=atan2(-r(1,2)/cos(rsa(i)*pi/180),r(2,2)/cos(rsa(i)*pi/180))*180/pi;

rsc(i)=atan2(-r(3,1)/cos(rsa(i)*pi/180),r(3,3)/cos(rsa(i)*pi/180))*180/pi;

RElbangle(i)=180-(acos(dot(yRUA(i,:),unit(WristR(i,:)-ElbowR(i,:))))*180/pi);

end

%Define the left upper arm coordinate system

token = 'yzx';

originLUA=ElbowL;

segmentOrigin = ElbowL;

[originLUA, xLUA, yLUA, zLUA] = ...

defineSegment(segmentOrigin,ShoulderL-ElbowL,WristL-ElbowL,token);

LUA = [xLUA,yLUA,zLUA];

for i=1:length(xLUA)

L=reshape(TOR(i,:), 3, 3)'* reshape(LUA(i,:), 3, 3);

Lsa(i)=-atan2(L(3,2),(L(2,2)^2+L(1,2)^2)^0.5)*180/pi;

Lsb(i)=atan2(-L(1,2)/cos(Lsa(i)*pi/180),L(2,2)/cos(Lsa(i)*pi/180))*180/pi;

Lsc(i)=-atan2(-L(3,1)/cos(Lsa(i)*pi/180),L(3,3)/cos(Lsa(i)*pi/180))*180/pi;

LElbangle(i)=180-(acos(dot(yLUA(i,:),unit(WristL(i,:)-ElbowL(i,:))))*180/pi);

end

2. Inter-repetition reliability

Inter-repetition reliability of the Azure Kinect and IMU systems across five repeated trials. For each participant, task, and joint angle, the intraclass correlation coefficient (ICC₂,₅) and coefficient of variation (CV%) were calculated from the five repeated measurements to assess the relative and absolute consistency of repeated trials within a single session. Values are presented for both the Azure Kinect and the IMU reference system.

**Supplementary Table S1.** Inter-repetition reliability (ICC₂,₅ and CV%) of the Azure Kinect and IMU systems across five repeated trials for each participant, task, and joint angle.

|  |  | |  | **AW** | **PC** | **HTM** | **CH** | **FR** | **UR** |
| --- | --- | --- | --- | --- | --- | --- | --- | --- | --- |
| **Shoulder FLX/EXT** | **Kinect** | **ICC (95%CI)** | | .990 (0.974-.997) | .933 (.846–.978) | .986 (.964–.995) | .984 (.963–.995) | .903 (.779–.968) | .981 (.956–.994) |
|  |  | **CV (%)** | | 11.04±4.5 | 14.29±7.06 | 8.09±2.22 | 6.71±4.98 | 12.23±5.51 | 8.41±4.59 |
|  | **IMU** | **ICC (95%CI)** | | .988 (.972-.996) | .992 (.983–.997) | .993 (.983–.998) | .989 (.975–.997) | .992 (.982–.998) | .992 (.982–.997) |
|  |  | **CV (%)** | | 6.69±6.40 | 11.27±6.71 | 4.92±3.17 | 5.95±8.17 | 6.84±4.25 | 6.26±3.94 |
| **Shoulder ABD/ADD** | **Kinect** | **ICC (95%CI)** | | .949 (.886–.984) | .932 (.825–.982) | .987 (.970–.996) | .977 (.945–.993) | .937 (.848–.979) | .906 (.777–.969) |
|  |  | **CV (%)** | | 15.76±5.72 | 24.49±9.19 | 12.00±1.01 | 12.74±12.87 | 16.99±8.08 | 11.96±8.16 |
|  | **IMU** | **ICC (95%CI)** | | .996 (.991–.998) | .979 (.954–.993) | .958 (.905–.986) | .998 (.996–.999) | .979 (.949–.994) | .939 (.854–.981) |
|  |  | **CV (%)** | | 6.47±5.71 | 18.24±8.18 | 17.59±13.13 | 8.76±12.03 | 17.25±7.18 | 18.02±15.70 |
| **Shoulder IR/ER** | **Kinect** | **ICC (95%CI)** | | .914 (.804–.972) | .981 (.943–.996) | .981 (.957–.994) | .964 (.917–.988) | .921 (.817–.974) | .966 (.923–.989) |
|  |  | **CV (%)** | | 15.68±7.66 | 18.62±8.99 | 13.72±7.48 | 15.13±8.77 | 20.33±7.45 | 14.89±5.43 |
|  | **IMU** | **ICC (95%CI)** | | .985 (.966–.995) | .834 (.624–.945) | .973 (.938–.991) | .981 (.956–.994) | .984 (.964–.995) | .969 (.931–.989) |
|  |  | **CV (%)** | | 5.74±3.24 | 23.19±8.39 | 14.22±7.05 | 15.99±7.07 | 12.12±7.45 | 14.59±12.78 |
| **Elbow EF/** | **Kinect** | **ICC (95%CI)** | | .907 (.786–.969) | .922 (.822–.975) | .842 (.643–.948) | .871 (.708–.957) | .968 (.924–.989) | .964 (.915–.988) |
|  |  | **CV (%)** | | 9.75±4.65 | 17.87±12.68 | 6.42±3.36 | 6.64±3.86 | 12.26±8.73 | 19.56±12.09 |
|  | **IMU** | **ICC (95%CI)** | | .991 (.979–.997) | .996 (.989–.998) | .992 (.981–.997) | .985 (.965–.995) | .982 (.959–.994) | .983 (.961–.994) |
|  |  | **CV (%)** | | 2.98±1.38 | 6.11±4.16 | 2.44±1.85 | 3.92±3.17 | 12.57±10.98 | 13.66±8.65 |
